# Molecular imaging of arterial fibroblast activation protein: association with calcified plaque burden and cardiovascular risk factors

**DOI:** 10.1007/s00259-023-06245-w

**Published:** 2023-05-06

**Authors:** Aleksander Kosmala, Sebastian E. Serfling, Kerstin Michalski, Thomas Lindner, Andreas Schirbel, Takahiro Higuchi, Philipp E. Hartrampf, Thorsten Derlin, Andreas K. Buck, Alexander Weich, Rudolf A. Werner

**Affiliations:** 1 Department of Nuclear Medicine, University Hospital Würzburg, Oberdürrbacher Str. 6, 97080 Würzburg, Germany; 2 Faculty of Medicine, Dentistry and Pharmaceutical Sciences, Okayama University, Okayama, Japan; 3 Department of Nuclear Medicine, Hannover Medical School, Hannover, Germany; 4 Internal Medicine II, Gastroenterology, University Hospital Würzburg, Würzburg, Germany; 5 NET-Zentrum Würzburg, European Neuroendocrine Tumor Society Center of Excellence (ENETS CoE), University Hospital Würzburg, Würzburg, Germany; 6 The Russell H Morgan Department of Radiology and Radiological Sciences, Johns Hopkins School of Medicine, Baltimore, MD USA

**Keywords:** [^68^ Ga]Ga-FAPI, Fibroblast activation protein, Atherosclerosis, Atherosclerotic plaque, Cardiovascular risk factors, Tumor burden

## Abstract

**Purpose:**

We aimed to assess prevalence, distribution, and intensity of in-vivo arterial wall fibroblast activation protein (FAP) uptake, and its association with calcified plaque burden, cardiovascular risk factors (CVRFs), and FAP-avid tumor burden.

**Methods:**

We analyzed 69 oncologic patients who underwent [^68^ Ga]Ga-FAPI-04 PET/CT. Arterial wall FAP inhibitor (FAPI) uptake in major vessel segments was evaluated. We then investigated the associations of arterial wall uptake with calcified plaque burden (including number of plaques, plaque thickness, and calcification circumference), CVRFs, FAP-positive total tumor burden, and image noise (coefficient of variation, from normal liver parenchyma).

**Results:**

High focal arterial FAPI uptake (FAPI +) was recorded in 64/69 (92.8%) scans in 800 sites, of which 377 (47.1%) exhibited concordant vessel wall calcification. The number of FAPI + sites per patient and (FAPI +)-derived target-to-background ratio (TBR) correlated significantly with the number of calcified plaques (FAPI + number: *r* = 0.45, *P* < 0.01; TBR: *r* =  − 0.26, *P* = 0.04), calcified plaque thickness (FAPI + number: *r* = 0.33, *P* < 0.01; TBR: *r* =  − 0.29, *P* = 0.02), and calcification circumference (FAPI + number: *r* = 0.34, *P* < 0.01; TBR: *r* =  − 0.26, *P* = 0.04). In univariate analysis, only body mass index was significantly associated with the number of FAPI + sites (*OR* 1.06; 95% CI, 1.02 − 1.12, *P* < 0.01). The numbers of FAPI + sites and FAPI + TBR, however, were not associated with other investigated CVRFs in univariate and multivariate regression analyses. Image noise, however, showed significant correlations with FAPI + TBR (*r* = 0.30) and the number of FAPI + sites (*r* = 0.28; *P* = 0.02, respectively). In addition, there was no significant interaction between FAP-positive tumor burden and arterial wall FAPI uptake (*P* ≥ 0.13).

**Conclusion:**

[^68^ Ga]Ga-FAPI-04 PET identifies arterial wall lesions and is linked to marked calcification and overall calcified plaque burden, but is not consistently associated with cardiovascular risk. Apparent wall uptake may be partially explained by image noise.

## Introduction

There is increasing evidence that fibroblast activation protein (FAP) orchestrates the development of atherosclerosis, by regulating the tight interaction of inflammatory response and fibrotic alterations [1]. For instance, macrophage-derived TNF-alpha in smooth muscle cells triggers FAP expression and is also linked to type I collagen breakdown in atherosclerotic fibrotic caps [2], leading to destabilization, cap rupture, and ultimately, acute coronary syndrome or myocardial infarction [3].

In recent years, plaque-based pathophysiological characteristics on a subcellular level have been visualized by various positron emission tomography (PET) agents, including inflammatory-targeting [^18^F]-fluorodeoxyglucose, the C-X-C motif chemokine receptor 4 PET probe [^68^ Ga]-PentixaFor, or microcalcification-directed 18F-sodium fluoride [4–6]. PET radiotracers targeting prostate specific membrane antigen (PSMA), however, could not establish a relevant link between uptake in the arterial tree and cardiovascular risk factors (CVRF), as the observed vessel wall signal was rather explained by image noise [7].

Despite its relevant role in the context of atherosclerosis [1], there is only limited data on the potential usefulness of FAP inhibitor (FAPI)-directed PET as a cardiovascular image biomarker [8]. As such, we aimed to characterize in vivo FAPI uptake in the arterial tree, along with established measures of arterial calcification [9, 10]. Similar to previous reports on PSMA-PET [7], we also aimed to investigate whether derived findings in the vessels may be explained by image noise. Last, previous work analyzed arterial wall uptake of patients affected with cancer [8, 11]. Thus, we also aimed to investigate whether FAPI uptake in major vessels may be associated with PET-avid tumor burden, thereby allowing to determine the role of FAPI PET as a cardio-oncology biomarker.

## Materials and methods

### Patient population

For this single-center retrospective analysis, we evaluated 69 patients affected with oncological diseases, which were referred for FAPI-directed PET/CT. Table 1 shows patients’ characteristics. Patients with available imaging and clinical data (including CVRF: history of smoking, arterial hypertension, hyperlipidemia, diabetes mellitus, body mass index (BMI), previous cardiovascular events, age > 60 years, and male gender) were included. Patients were then classified as low (0–1 CVRF), intermediate (2–3 CVRF), and high-risk (≥ 4 CVRF). Additionally, cardiovascular risk stratification according to the ESC SCORE2/SCORE2-OP risk chart was performed [12]. Exclusion criteria were a history of chemotherapy within 4 weeks prior to the scan and known vasculitis or systemic inflammatory disease. Due to the retrospective nature of this study, the local institutional review board (No. #20,210,415 02) waived the need for further approval. Parts of this cohort have been investigated in previous studies [13–18]. However, none of these studies evaluated vessel wall associated FAPI uptake.

**Table 1:** Patients’ characteristics (*n* = 69)

Age^†^	63 ± 11 years (38–89)
Gender (female) ‡	26/69 (37.7)
BMI (kg/m^2^) ^†^	25.4 ± 4.3 (16.8–43.3)
Tumor entity^‡^	
HNSCC	27/69 (39.1)
NEN	18/69 (26.1)
Lung	8/69 (11.6)
HCC	6/69 (8.7)
Other*	10/69 (14.5)
Cardiovascular risk factors^‡^	
Male gender	43/69 (62.3)
Age > 60 years	41/69 (59.4)
Hypertension	35/69 (50.7)
History of smoking	27/69 (39.1)
Diabetes mellitus	8/69 (11.6)
Hypercholesterinemia	7/69 (10.1)
Prior cardiovascular event	7/69 (10.1)
Laboratory values^†^	
CRP	2.4 ± 5.5 (0–33.3)
WBC count	8.0 ± 2.9 (3.6–20.4)
GFR	84.1 ± 21.6 (10–124.4)
Cardiac medication^‡^	
ARB/ACE-inhibitor	22/69 (31.9)
Beta-blocker	17/69 (24.6)
Statins	14/69 (20.3)
Aspirin	10/69 (14.5)

^†^Values are mean ± standard deviation, with range in parenthesis. ^‡^Values are number of patients, with percentages in parenthesis. *Including pancreatic duct adenocarcinoma (*n* = 3), colon adenocarcinoma (*n* = 2), sarcoma (*n* = 1), adrenocortical carcinoma (*n* = 1), solitary fibrous tumor (*n* = 1), gastrointestinal stroma tumor (*n* = 1), phosphaturic mesenchymal tumor (*n* = 1). *BMI*, body mass index. *HNSCC*, head and neck squamous cell carcinoma; *NEN*, neuroendocrine neoplasm; *HCC*, hepatocellular carcinoma. *CRP*, C-reactive protein. *WBC*, white blood cell. *GFR*, glomerular filtration rate. *ARB*, angiotensin-receptor-blocker. *ACE*, angiotensin-converting enzyme

### Imaging procedure

[^68^ Ga]Ga-FAPI-04 was synthesized and labeled as specified in [19]. All scans were acquired using a Siemens Biograph mCT 64 or 128 (Siemens Healthineers, Erlangen, Germany). The median injected activity of [^68^ Ga]Ga-FAPI-04 was 148 MBq (range, 79–168 MBq). Whole body PET scans (range, vertex to mid thighs) were initiated after an approximate time of 60 min post injection (parameters: mCT 64, 2–3 min/bed position/mCT 128, continuous bed motion at 1.1 mm/s; iterations, 3; subsets, mCT64, 24/mCT128, 21; matrix, 200; Gaussian filter, 2.0 mm). Low dose CT scans were then performed for attenuation correction (parameters: tube voltage, 120 kV; activated automatic tube current modulation, reference at 35 mAs; pitch, 0.8; collimation, 64/128 × 0.6 mm; reconstructed axial slice thickness, 3.0–5.0 mm) [13, 18].

### Image evaluation

Commercial software (syngo.via, version VB60A; Siemens Healthineers, Erlangen, Germany) installed on a dedicated workstation was used to analyze PET, CT, and fused PET/CT images. A board-certified radiologist with 9 years of experience in oncologic and cardiovascular imaging (A. K.) evaluated the following seven large artery segments for signs of calcified plaques and/or high focal vessel wall associated radiotracer uptake: carotid arteries, ascending aorta, aortic arch, descending thoracic aorta, abdominal aorta, iliac arteries, and femoral arteries, while paired arteries were then evaluated as one single vessel segment on further analyses. In inconclusive cases of arterial wall uptake, two board-certified nuclear medicine physicians were consulted (S.E.S., R.A.W.).

### Radiotracer uptake

PET images were visually assessed for radiotracer uptake, and fused images were utilized to accurately localize the corresponding areas in relation to the vessel wall and vessel wall-associated calcifications [5]. Relevant focal arterial wall FAPI uptake (FAPI +) was defined as a minimum target-to-background ratio (TBR) of 1.6, in accordance with previous studies on FAPI PET in the context of cardiovascular imaging [8]. Individual 3-dimensional volumes of interest (10 mm in diameter) were placed around corresponding sites on PET, which then provided the maximum standardized uptake (SUV_max_). Blood-pool SUV_mean_ was derived from the mean of three regions of interest of at least 10 mm diameter on non-adjacent slices within the mid lumen of the superior vena cava. TBRs were calculated as SUV_max_ in FAPI + sites divided by the blood-pool SUV_mean_ [5].

For each patient, an additional PET-based analysis of FAP-positive total tumor burden was performed as described in [15], and SUV_max_, peak SUV (SUV_peak_), sum of total tumor volume (TV, in cm^3^), and fractional tumor activity (FTA = TV * SUV_mean(tumor)_) were recorded.

### Calcified plaque analysis

Calcified plaques (CP +) were defined as high-density vessel wall areas with an attenuation > 130 Hounsfield units on CT images [7]. For each CP + lesion, we recorded maximum calcification thickness in mm. Additionally, the maximum circumferential calcification extent was visually graded on a 4-point scale: 1, 1–25% of the vessel wall circumference; 2, 26–50% of the vessel wall circumference; 3, 51–75% of the vessel wall circumference; and 4, > 75% of the vessel wall circumference [7, 9].

### Image noise analysis

Image noise was determined using the coefficient of variation (CoV) within normal liver parenchyma [6]. After placing a 3 cm region of interest in an area of uniform uptake in the right liver lobe, the CoV was determined as the ratio of standard deviation to SUV_mean_.

### Statistical analysis

Categorical variables are reported as absolute and relative frequencies, while continuous variables are presented as mean ± SD. The Kolmogorov–Smirnov test was applied to investigate a normal distribution of the data. Normally distributed data are presented as mean ± SD, otherwise as median and range. The *t* test was used for group-comparisons of parametric data. The Mann–Whitney rank sum test was used for group-comparisons of non-parametric data and the Spearman correlation coefficient was used for correlative analyses (with correction for outliers using the ROUT-method). Comparisons between CVRF-groups were performed using the Kruskal–Wallis test with Dunn’s correction for multiple comparisons. To predict the number of FAPI + and/or CT + lesions by CVRF, age, and gender, negative binomial regression was used due to overdispersion. Linear regression was used to evaluate the relationship between CVRF, age, gender, and FAPI + TBR. A *P*-value < 0.05 was considered statistically significant. Statistical analyses were performed in R (version 3.6.1, R Core Team, 2019) with package MASS (7.3–51.4) and GraphPad Prism Version 9.3.1 (GraphPad Prism Software, La Jolla, CA, USA) [20, 21].

## Results

### Vessel wall FAPI uptake

Detailed information on arterial wall FAPI uptake is listed in Table 2. Focally increased vessel wall associated FAPI uptake was seen in 64/69 (92.8%) scans in a total of 800 sites. The prevalence of uptake sites was highest in the abdominal aorta, followed by the descending thoracic aorta and the iliac arteries. Overall, SUV_max_ was 2.9 ± 0.9 ranging from 1.3 to 7.8, with highest values recorded in the descending thoracic aorta. Mean blood pool SUV was 1.2 ± 0.2 (range, 0.7–1.9). Mean TBR was 2.5 ± 0.6 (range, 1.6–5.8), with highest values again documented in the descending thoracic aorta.

**Table 2:** Vascular FAPI uptake

	Carotid arteries	Ascending aorta	Aortic arch	Descending thoracic aorta	Abdominal aorta	Iliac arteries	Femoral arteries	All vessels
Number (%) of patients with vessel wall associated FAPI uptake	16 (23.2)	12 (17.4)	42 (60.9)	58 (84.1)	61 (88.4)	45 (65.2)	25 (36.2)	64 (92.8)
Total number of uptake sites	24	18	81	294	239	105	39	800
Sites with concomitant calcification *n* (%)	21 (87.5)	7 (38.9)	38 (46.9)	70 (23.8)	143 (59.8)	71 (67.6)	27 (69.2)	377 (47.1)
SUV_max_								
Mean ± SD	2.6 ± 0.6	2.9 ± 0.9	2.9 ± 0.8	3.1 ± 0.9	2.9 ± 0.9	2.9 ± 0.9	2.5 ± 0.6	2.9 ± 0.9
Range	1.8–4.4	1.7–5.6	1.7–6.5	1.5–7.8	1.3–7.1	1.5–5.4	1.4–3.9	1.3–7.8
TBR								
Mean ± SD	2.2 ± 0.5	2.5 ± 0.6	2.5 ± 0.6	2.6 ± 0.6	2.5 ± 0.7	2.5 ± 0.7	2.3 ± 0.5	2.5 ± 0.6
Range	1.6–3.8	1.6–3.9	1.6–4.6	1.6–5.0	1.6–5.8	1.6–5.8	1.6–3.4	1.6–5.8
SUV_mean blood-pool_								
Mean ± SD								1.2 ± 0.2
Range								0.7–1.9

*FAPI*, fibroblast activation protein inhibitor; *SUV*, standardized uptake value; *TBR*, target-to-background ratio; *SUV*_mean blood pool_, mean standardized uptake value derived from the mean of three regions of interest of at least 10 mm diameter on non-adjacent slices within the mid lumen of the superior vena cava serving as reference tissue for TBR calculations

### Calcified plaque burden

A total of 64/69 (92.8%) patients showed arterial vessel wall calcifications, in a total of 851 sites. The highest number of calcified plaques was recorded in the abdominal aorta, followed by the iliac arteries and the descending thoracic aorta. Mean calcification circumference score was 1.6 ± 0.9 (range, 1–4). Mean thickness of recorded calcified plaques was 2.8 ± 1.2 mm, ranging from 1 to 8 mm. Table 3 shows detailed information on calcified plaques of arterial vessel walls.

**Table 3:** Distribution of vascular calcification

	Carotid arteries	Ascending aorta	Aortic arch	Descending thoracic aorta	Abdominal aorta	Iliac arteries	Femoral arteries	All vessels
Number (%) of patients with vessel wall calcification	30 (43.5)	9 (13.0)	43 (62.3)	38 (62.3)	57 (55.1)	54 (78.3)	36 (52.2)	64 (92.8)
Total number of calcified sites	52	9	70	175	272	194	79	851
Calcification circumference score								
Mean ± SD	2.1 ± 1.0	1.2 ± 0.4	1.1 ± 0.3	1.2 ± 0.5	1.7 ± 1.0	1.9 ± 1.0	1.6 ± 0.8	1.6 ± 0.9
Range	1–4	1–2	1–2	1–4	1–4	1–4	1–4	1–4
Calcification thickness (mm)								
Mean ± SD	3.0 ± 1.2	2.8 ± 1.6	3.0 ± 1.4	2.4 ± 1.1	2.8 ± 1.3	2.9 ± 1.1	2.8 ± 1.0	2.8 ± 1.2
Range	1–6	1–6	1–7	1–6	1–8	1–5	1–5	1–8

### Number of FAPI + sites and TBR correlated with CP + sites, calcified plaque thickness, and calcification score

On a per-patient level, 61/69 (88.4%) were FAPI + /CP + scans. A total of 3/69 (4.3%) scans were FAPI-/CP + , 3/69 (4.3%) were FAPI + /CP-, and the remaining 2/69 (2.9%) were classified as FAPI-/CP-.

On a per-segment basis, out of 483 segments, 283 (58.6%) were FAPI + , and 267 (55.3%) were CP + . Out of 283 FAPI + segments, 205 (72.4%) were also CP + , while out of 200 FAPI- segments, 62 (31.0%) were CP + . A total of 138/483 (28.6%) segments did not show any FAPI + focal vessel wall uptake or calcified plaques.

**Fig. 1:**
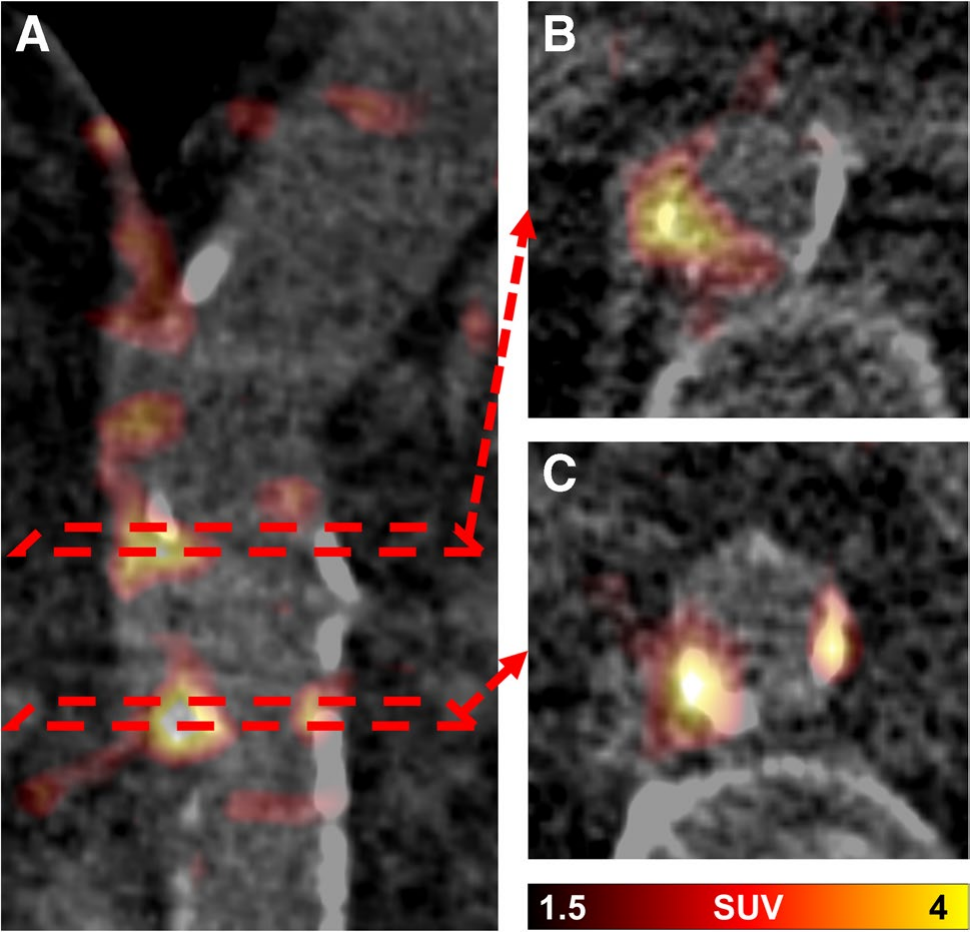
Fused axial [^68^ Ga]Ga fibroblast activation protein inhibitor (FAPI) PET/CT images of the aorta of a 72-year-old man: coronal PET/CT image (**A**) and corresponding axial PET/CT slices (**B** and **C**). In **B**, radiotracer uptake in the vessel wall coincides with and exceeds vessel wall calcification, while other calcifications show no increased tracer uptake. In **C**, FAPI uptake is colocalized with vessel wall calcifications

**Fig. 2:**
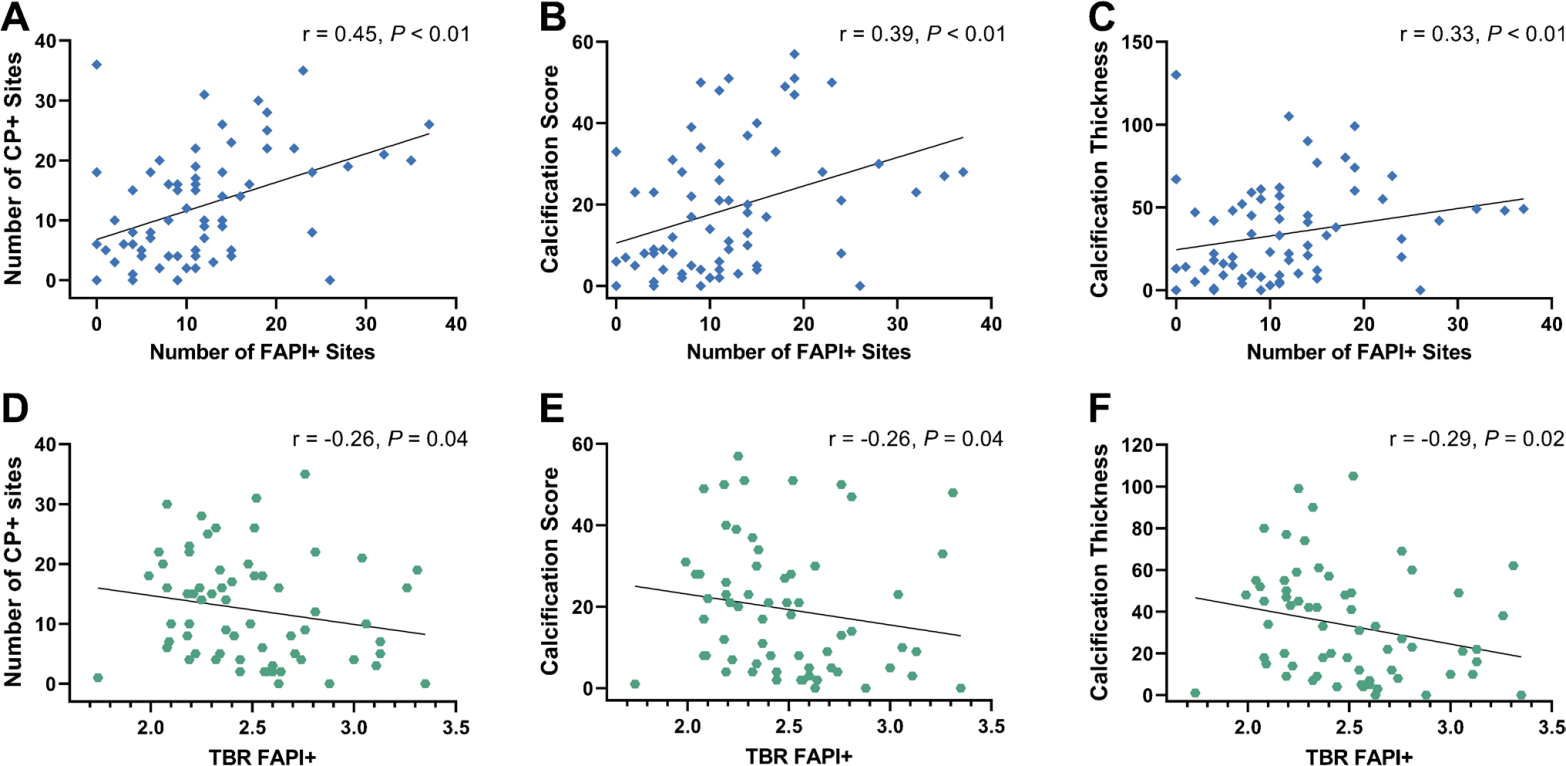
Associations of numbers of fibroblast activation protein inhibitor uptake (FAPI +) sites (upper row) and tracer accumulation defined as target-to-background ratio (TBR; lower row) with parameters of calcified plaque (CP +) burden: number of CP + sites (**A** and **D**), calcification score (**B** and **E**), and calcification thickness (**C** and **F**). For **B**, **C**, **E**, and **F**, cumulative values were used

On a per-lesion basis, 377/800 (47.1%) FAPI uptake sites exhibited concordant vessel wall calcification (Fig. 1), which translates to 44.3% (377/851) of CP + sites with increased focal FAPI uptake. Consistently, the number of FAPI + sites per patient correlated significantly with the number of CP + sites (*r* = 0.45, *P* < 0.01), calcified plaque thickness (*r* = 0.33, *P* < 0.01), and calcification score (*r* = 0.39, *P* < 0.01; Fig. 2). FAPI + TBR also showed significant correlations with the number of CP + sites (*r* =  − 0.26, *P* = 0.04), calcified plaque thickness (*r* =  − 0.29, *P* = 0.02), and calcification score (*r* =  − 0.26, *P* = 0.04; Fig. 2).

### Numbers of FAPI + sites are associated with BMI and CP + sites are linked to CVRFs

On average, patients displayed a median of 2 CVRF (range, 0–6). Median ESC risk chart score was 12 (range, 1–47), and respective correlations with the number of FAPI + sites, FAPI + TBR, and CP + were as follows: number of FAPI + sites (*r* = 0.37, *P* = 0.02), FAPI + TBR (*r* = 0.15, *P* = 0.38), and CP + (*r* = 0.70, *P* < 0.01).

**Fig. 3:**
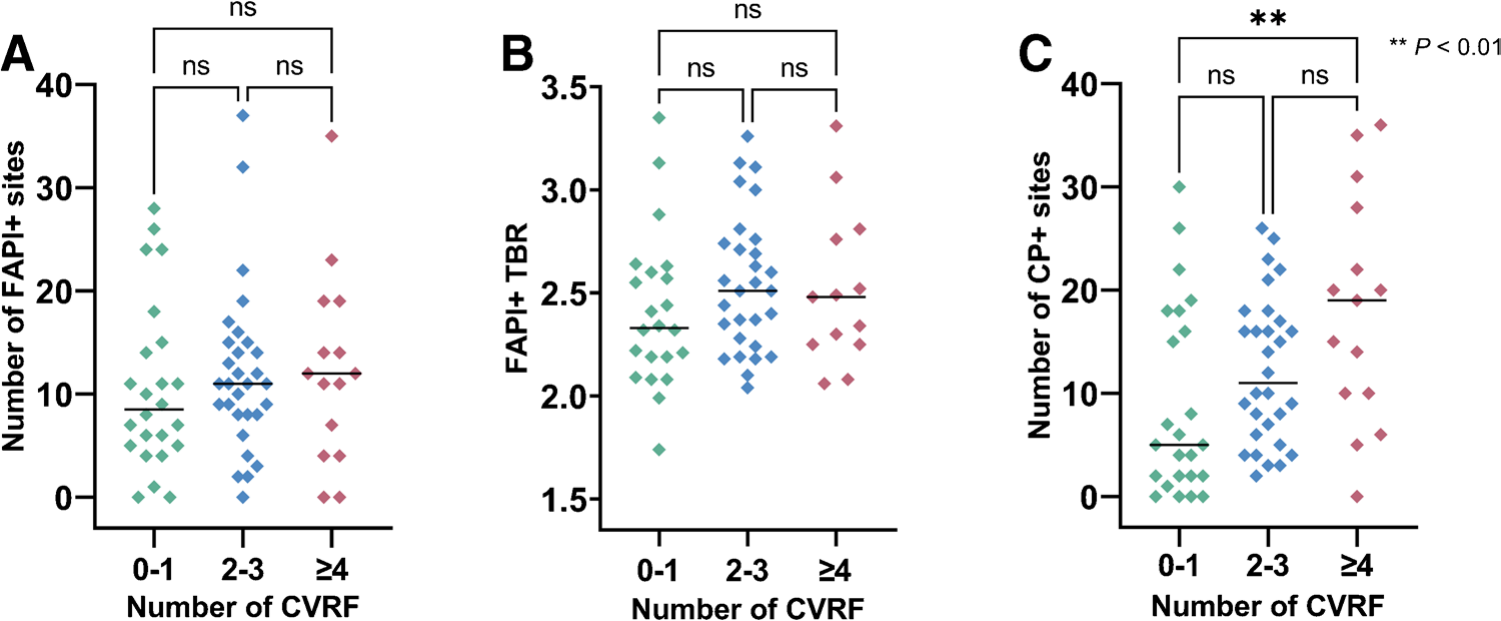
Scatter plots showing the number of high fibroblast activation protein inhibitor uptake sites (FAPI +) (**A**), the mean target-to-background ratio (TBR) in FAPI + sites (**B**), and the number of calcified plaque sites (CP +) (**C**), grouped by the number of cardiovascular risk factors (CVRF). Horizontal black line indicates median. The numbers in respective CVRF subgroups vary depending whether uptake on PET (for both number of FAPI + sites and FAPI + TBR) or CP + sites were assessable

**Fig. 4:**
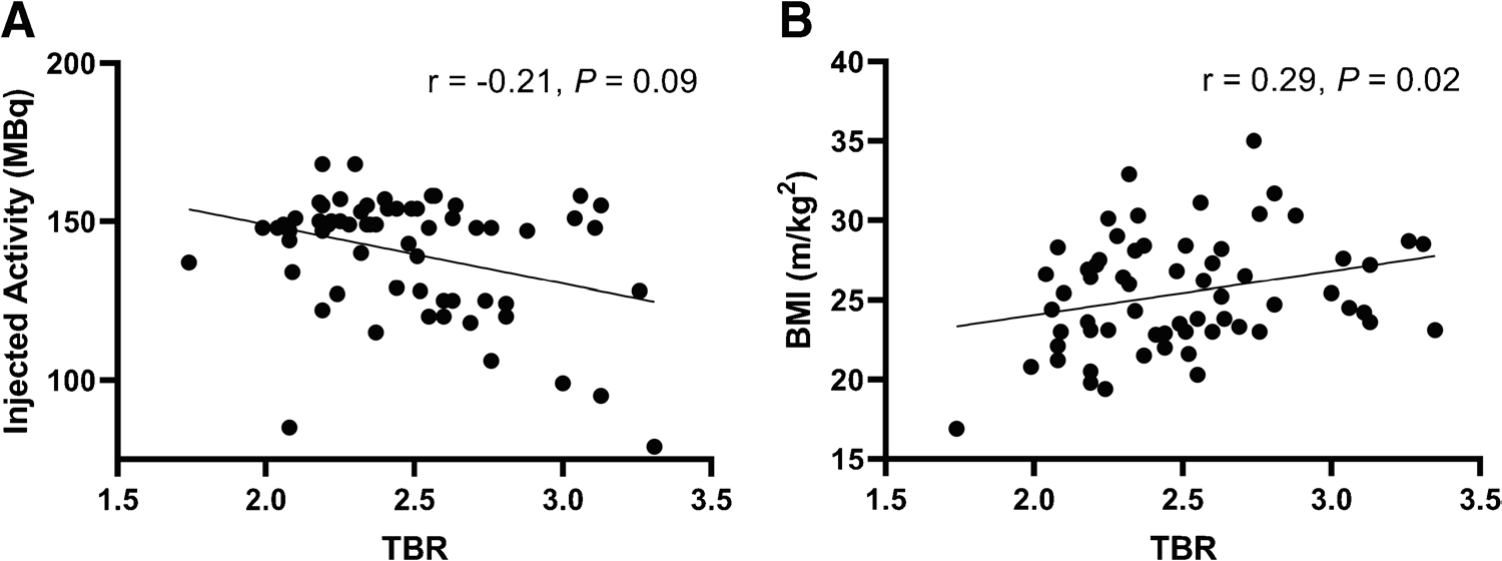
Associations of tracer accumulation defined as target-to-background ratio (TBR) and parameters contributing to image noise, including injected activity (**A**) and body mass index (BMI) (**B**). Higher injected activities were linked to less uptake. Overweight patients had higher TBR, which can also be explained by image noise [27]

The number of FAPI + sites (*r* = 0.09, *P* = 0.45) and FAPI + TBR (*r* = 0.07, *P* = 0.57) did not show any significant correlation with the number of CVRF. Groupwise analysis of low, intermediate, and high-risk patients showed no significant differences between groups for number of FAPI + sites and FAPI + TBR (Fig. 3). In univariate regression analysis, the numbers of FAPI + sites were significantly associated with BMI (*OR* 1.06; 95% CI, 1.02–1.12; *P* < 0.01), but not any other CVRF, age, or injected activity, thereby limiting the number of significant predictors for multiple regression. Comparable results were recorded for FAPI + TBR and CVRF on regression analyses, with a trend towards significance for BMI in univariate analysis (*OR* 1.02; 95% CI 1.00–1.04; *P* = 0.09; female gender, *OR*, 0.78; 95% CI 0.66–0.93, *P* < 0.05). In addition, BMI also achieved significant correlations with TBR (*r* = 0.29, *P* = 0.02), while injected activity trended inversely towards significance (*r* =  − 0.25, *P* = 0.05; Fig. 4).

Unlike findings on PET, the number of CVRFs correlated significantly with the number of CP + sites (*r* = 0.37, *P* < 0.01). Groupwise analysis also revealed significantly more CP + sites in high-risk patients when compared with low-risk patients (*P* < 0.01; Fig. 3). In univariate regression analysis, arterial hypertension (*OR* 1.63; 95% CI, 1.09–2.32; *P* = 0.02) and age (*OR* 1.04; 95% CI, 1.03–1.06; *P* < 0.01) showed significant associations with the number of CP + sites. In multivariate analysis, only age (*OR* 1.04; 95% CI, 1.02–1.06; *P* < 0.01) was significantly associated with the number of CP + sites, with a trend towards significance for hypertension (*OR* 1.40; 95% CI, 0.99–1.98; *P* = 0.06).

### Laboratory markers of inflammation were not associated with the number of FAPI + sites, FAPI + TBR, and number of CP + sites

White blood cell count (WBC) and C-reactive protein (CRP) did not correlate significantly with the number of FAPI + (WBC: *r* = 0.03, *P* = 0.79; CRP: *r* =  − 0.06, *P* = 0.65) or CP + (WBC: *r* = 0.12, *P* = 0.35; CRP: *r* = 0.01, *P* = 0.97) sites. Also, FAPI + TBR was not correlated with WBC (*r* =  − 0.03, *P* = 0.82) or CRP (*r* =  − 0.08, *P* = 0.56).

### Image noise correlates with BMI, injected activity, number of FAPI + sites, and TBR

Mean image noise assessed by normal liver parenchymal CoV was 37.1 ± 11.2 (range, 16.7–63.3). CoV showed significant correlations with BMI (*r* = 0.47; *P* < 0.01) and injected activity (*r* =  − 0.25; *P* = 0.04). In addition, FAPI + TBR (*r* = 0.30) and the number of FAPI + sites (*r* = 0.28) also correlated significantly with CoV (*P* = 0.02, respectively).

### Arterial FAPI uptake does not show relevant associations with tumor burden

In 55/69 (79.7%) patients, uptake in tumor sites was recorded. Descriptive statistics of tumor-associated radiotracer uptake were as follows: SUV_max_ (median, 13.8; range 4.25–34.4), SUV_peak_ (median, 9.68; range, 1.73–27.0), TV (median, 11.6; range, 1.82–432), and FTA (median, 6.36; range, 6.36–3217). The number of FAPI + vessel wall sites did not correlate significantly with tumor SUV_max_ (*r* =  − 0.08, *P* = 0.56), tumor SUV_peak_ (*r* =  − 0.15, *P* = 0.29), TV (*r* =  − 0.07, *P* = 0.62), or FTA (*r* =  − 0.13, *P* = 0.369). Also, there was no significant correlation between FAPI + vessel wall TBR and tumor SUV_max_ (*r* = 0.22, *P* = 0.13), tumor SUV_peak_ (*r* = 0.16, *P* = 0.26), TV (*r* = 0.05, *P* = 0.72), or FTA (*r* = 0.15, *P* = 0.29). When comparing patients with vs. without FAPI positive tumor burden, no significant difference was observed in the number of FAPI + vessel wall sites (with tumor burden: median, 11; range, 0–35 vs. without tumor burden: median, 13; range, 4–37; *P* = 0.08) or FAPI + vessel wall TBR (with tumor burden: median, 2.49; range 1.74–3.35 vs. without tumor burden: median, 2.34; range, 2.06–2.64; *P* = 0.14).

**Fig. 5:**
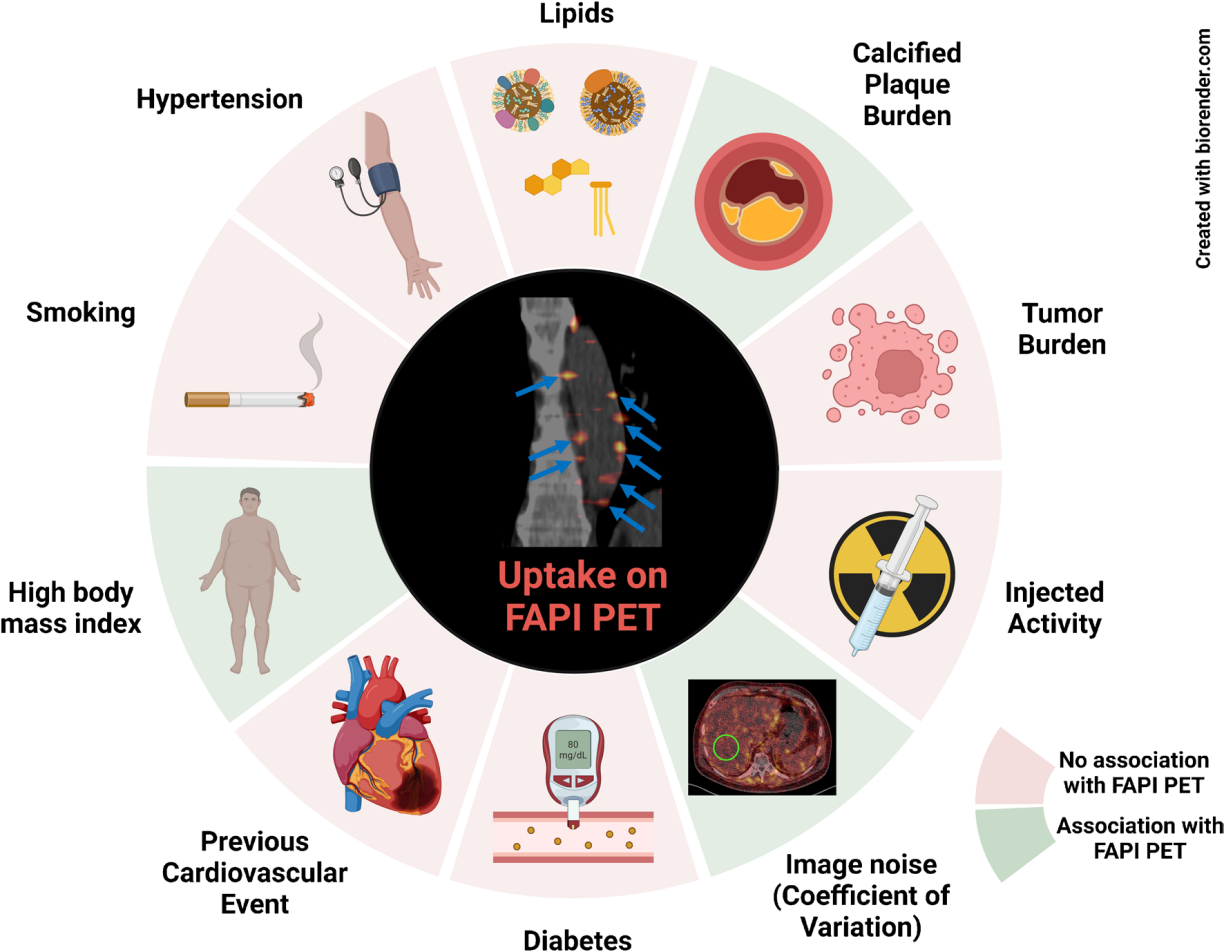
PET-based FAPI uptake in the arterial tree is linked to plaque burden, but is not consistently associated with cardiovascular risk, which may be explained by image noise. *PET*, positron emission tomography. *FAPI*, fibroblast activation protein inhibitor

A comprehensive overview of our results is shown in Fig. 5.

## Discussion

In oncology patients scanned with [^68^ Ga]Ga-FAPI-04, we observed focally increased arterial FAPI uptake in more than 92% of the scans and in a total of 800 sites, while vessel wall calcifications were recorded in 851 instances. Of note, in less than 50%, FAPI-positive arterial wall sites also exhibited concordant vessel wall calcification. Describing varying facets of total calcified atherosclerotic plaque burden, the number of FAPI + sites per patient and FAPI + TBR correlated significantly with the number of CP + sites, calcified plaque thickness, and calcification score. Despite those associations between morphology-based alterations and functional imaging, only the number of CP + sites was associated with CVRFs (including age and arterial hypertension), but not the FAPI PET signal. In addition, we recorded significantly more CP + sites in high-risk (≥ 4 CVRF) relative to low-risk patients (0–1 CVRF), while such group-wise comparisons revealed no significant differences for the number of FAPI + sites and FAPI + TBR. As such, FAPI-targeting PET may be less suitable as a cardiovascular image biomarker in patients with limited number of CVRF. In addition, the observed findings may also be explained by image noise, as substantial correlative indices were observed between TBR and BMI, but also for BMI and the number of FAPI + sites in univariate analysis. Further supporting the notion that observed findings on PET are most likely caused by image noise, CoV also demonstrated significant correlations with TBR and the number of FAP + sites. Last, there were no relevant associations between vessel wall-derived FAPI uptake and FAPI-avid tumor burden and thus, FAPI PET may be also less useful in a cardio-oncology setting [22].

### Apparent arterial FAPI uptake is associated with calcified plaque burden, but is not consistently associated with cardiovascular risk

On a subcellular level, FAP mediates the development of atherosclerosis, by regulating inflammatory response (involved in rupture of plaques) and fibrotic remodeling (as a mediator of plaque stability) [1]. As such, using a recently introduced FAP-targeted PET agent initially developed for patients affected with oncological diseases, we investigated a large cancer cohort imaged with [^68^ Ga]Ga-FAPI-04 to perform an in-depth analysis of plaque burden with radiotracer accumulation in the arterial tree. First, almost half of the FAPI uptake sites demonstrated concordant vessel wall calcification and this low concordance rate may be explained by the fact that large arterial plaques with dense calcification are mostly stable plaques without significant risk of rupture, while FAP expression may be more likely related to unstable plaques [2, 23]. We also observed an inverse correlation of FAPI + TBR with the number of CP + sites. Comparable findings have been previously described by a study on 41 cancer patients scheduled for FAPI-directed PET, also with lower degree of calcification tightly linked to an increasing background-corrected uptake in arterial sites of disease [8]. Relative to the degree of calcification, however, CP + sites are also characterized by circumferential calcification extent and plaque thickness, with the latter parameter linked to a higher prevalence of intraplaque hemorrhage [24]. Thus, relative to previous work only focusing on degree of calcification [8], we also investigated both circumferential calcification and plaque thickness and again, observed inverse correlations with FAPI + TBR. The higher FAPI uptake in none or mildly calcified plaques may be partially explained by an ongoing plaque stabilization [1], leading to an early termination of atherosclerosis progression prior to development to severely calcified plaques. In addition, an aggregation of two CVRFs is already associated with an increased 10-year cardiovascular mortality [25] and thus, we grouped our cohort into low (0–1 CVRF), intermediate (2–3 CVRF), and high-risk patients (≥ 4CVRF). When compared with low-risk individuals, groupwise analysis then showed significant differences for high-risk patients for the number of CP + sites and the number of CVRFs correlated with the number of CP + sites. This link between risk factors and arterial calcification matches well with existing literature [26]. However, this association was not observed for FAPI + sites or TBR. As such, given our and previous work investigating minimum 2 CVRFs [8], one may speculate whether the derived signal in the arterial tree may be particularly useful for patients with cumulative exposure to CVRFs.

### Apparent arterial FAPI uptake may partially be explained by image noise

Image noise on PET is tightly linked to obesity, as a BMI range of 30 to 45 is associated with an increase in noise by almost 50% [27]. In patients with prostate cancer imaged with PSMA PET, subjects with higher BMI exhibited increased PSMA expression in the arterial tree, supporting the notion that uptake in the vessels was partially noise-related [7]. Of note, similar to previous work [7], we also observed an increased arterial FAPI uptake in patients with higher BMI, thereby explaining the missing correlations between CVRFs and radiotracer accumulation in our study. Therefore, while usually higher TBR-values suggest good image contrast, in this instance, the positive correlation of FAPI + TBR and CoV (as a marker for image noise) may conceivably be caused by noise-mediated clustering of activity on PET images as has been reported previously [7]. In addition, system-based correlations between CoV and FAPI signal in the vessels also reached significance, further supporting the notion that findings in the arterial tree are most likely caused by image noise. Second-generation, 18F-labeled radiotracers targeting fibroblasts may overcome this issue due the higher image quality when compared to Gallium-68 [28].

### Apparent arterial FAPI uptake is not linked to systemic markers of inflammation or FAPI-avid tumor burden

In contrast to prior studies reporting on an association between arterial wall [^18^F]-FDG uptake and inflammatory activity [29, 30], we did not observe a similar association between markers of systemic inflammation (CRP and WBC) and FAPI uptake in our current work. Although FAP may orchestrate the development of atherosclerotic plaques by mediating between the local inflammatory response and fibrotic alterations [1], our results indicate that arterial wall FAPI uptake is not an indicator of systemic inflammation.

Last, in cancer patients, myocardial FAPI PET was also associated with CVRFs and metabolic disease, in particular in subjects with focal uptake pattern in the heart [11]. We could not establish any relevant associations between uptake in the arterial tree and tumor sites and thus, FAPI signal may rather not be useful as a cardio-oncology biomarker in the context of atherosclerosis.

### Study limitations

Several limitations have to be considered, including the retrospective design and the limited number of patients. Nonetheless, we report on the largest cohort to determine FAP upregulation in CP sites or to define the role of FAPI PET as a cardiovascular biomarker. In addition, a whole-vessel-analysis or most-diseased-segment-analysis could be helpful to reduce impact of image noise. Moreover, the high number of patients with vessel calcifications as well as the restriction of our cohort to oncologic patients could limit the generalizability of our results. Furthermore, we did not investigate hard cardiovascular endpoints, such as cardiovascular death or acute myocardial infarction. Additionally, although linear regression correlations presented in this work show significant *P*-values, the wide dispersion of data along the regression line is reflected in mostly moderate r-values. In addition, we acknowledge that the measured CoV in our current work was higher than suggested by the EARL guidelines for [^18^F]-FDG [31] but extrapolation to a different radiotracer (FAPI) and radionuclide (Gallium-68) may be made with caution. Moreover, although coronary arteries are directly involved in the pathogenesis of myocardial infarction, we did not analyze them due to their small caliber and the lack of dedicated ECG-triggered and contrast enhanced coronary CT angiography scans. Further validation of atherosclerosis may also include head-to-head comparisons with 18F-labeled sodium fluoride [32, 33]. Last, negative findings in this study may also be explained by the fact that average diameter of the vessel wall is below the spatial resolution of the clinical PET system and thus, adding to partial volume effects [34] — a phenomenon that may be even more pronounced for a 68 Ga-labeled PET agent. Novel whole body PET systems provide increased sensitivity and thus, may overcome this issue, in particular for vessel wall analyses [6].

## Conclusion

In a cancer cohort scanned with [^68^ Ga]Ga-FAPI-04, we observed focal tracer accumulation in more than 92% in 800 sites. Investigating varying characteristics of plaque burden, the number of FAPI + sites per patient and FAPI + TBR correlated significantly with the number of CP + sites, calcified plaque thickness, and calcification score. Despite those associations between morphology-based alterations and functional imaging, FAPI + sites did not consistently associate with CVRFs. Results may in part be explained by image noise. Last, no link could be established by FAPI + TBR and FAPI-avid tumor burden.

## Data Availability

The datasets generated and analyzed during the current study are available from the corresponding author upon reasonable request.
